# The League of Mercy

**Published:** 1901-07-27

**Authors:** 


### THE INSTITUTIONAL WORKSHOP.

### THE LEAGUE OF MERCY.

GARDEN PARTY AT PADDINGTON.

At the invitation of Lady Burdett, a considerable number of the leading residents of Paddington accepted her hospitality at The Lodge, Porchester Square, last Thursday afternoon, the special object of the gathering being to enlist sympathy and support in the district on behalf of the League of Mercy. The guests were received by Sir Henry and Lady Burdett, and among the company were the Mayor of Paddington, Sir John Aird, Bart., M.P., and Lady Aird; Sir G. Fardell, M.P.; Judge Francis Bacon, Sir Fitzroy and Lady Maclean, Sir James Vaughan, General Sir Julius Raines, K.C.B., and Lady Raines; Sir Lacy Robinson, K.C.B., and Miss Robinson; Lady Cloete, Surg.-Gen. and Mrs. Muir, Mr. R. Anderson, C.B.; Deputy Surg.-Gen. and Miss Farquhar, Col. Hughes, Mr. H. A. Harben, Mrs. Adler, Rev. Prebendary Ridgeway, Mrs. Cooper, and Mr. and Mrs. Metcalfe.

Sir Henry Burdett, K.C.B., described the purposes of the League, which, he said, was established by the King by Royal Charter, with the personal co-operation of her late Majesty Queen Victoria, with the object of enabling the humblest citizen of London to give regularly any sum, however small, to the London hospitals, under a guarantee that every penny of it would go, without deduction, directly to the relief of the sick and suffering. The war in South Africa must in due course terminate, but London's war with disease and death in the cause of sickness and suffering must continue so long as the metropolis of the Empire existed. That battle, he pointed out, though thanks in large measure to Lord Lister's great discovery it was waged with ever-increasing success by the medical practitioners and managers of the London hospitals, was increasingly costly because it was increasingly successful; and, as the interests of the richest as of the poorest citizens demanded that every means which science could evolve should be brought to bear to the saving of suffering humanity, it was the privilege of all to cheerfully contribute something regularly to the hospitals. He next showed how every breadwinner, when illness or accident may render it necessary for him to seek the aid of a great London hospital, gains through the development in modern hospital treatment sixteen clear working days as compared with twenty years ago, and that 98,000 in-patients were treated in the hospitals and medical charities of London last year. The solid benefit in pounds sterling resulting from the efficiency of such institutions to-day, as compared with ten decades ago, may be realised from the fact that the improvements in treatment, by shortening the days of residence in a hospital, have added over a million of working days each year to the earning powers of the artisans of London. Sir Henry proceeded to emphasise the advantages derived by all classes from the work of the hospitals. It was true that, thanks to the medical profession, trained nurses for the sick, and the district or Queen's nurses for the poor, both the wealthy and the humble had a reasonable provision in their own homes in cases of ordinary illness; but in the day of serious illness they were dependent upon the direct aid which they derived from the hospitals. The League of Mercy afforded them an opportunity of doing their part, not merely as an act of self-preservation, but also as a thankoffering for the benefits they obtained. The League of Mercy was the machinery by which this could be done. It was organised by his Majesty the King as a branch of the Prince of Wales's Fund for the benefit of the London hospitals, which was the object the late beloved Queen Victoria selected out of four thousand proposals as the one nearest to her heart to commemorate her Diamond Jubilee. A donor of a shilling could become an associate of the League; and, as an illustration of the possibilities of the organisation in obtaining the mites of the multitude, Sir Henry mentioned that one lady had collected £20 nearly all in coppers, and largely in farthings, from the poor people in her district.

Sir John Aird, who wore the handsome chain appertaining to the office of the Mayor of Paddington, and apologised for not also wearing his robes, on account of the great heat, moved the following resolution:—"As citizens of Paddington it is essential that all classes should make it their business to understand the objects and work of the League of Mercy, his Majesty the King being the Sovereign of the Order, and the humblest being able through it to render a measure of personal service to the direct relief of sickness and suffering." In the course of an earnest appeal to his fellow citizens, Sir John dwelt on the vast amount of good effected by the Prince of Wales's Hospital Fund, and strongly advised his hearers, alike in their own interests and in the interests of suffering humanity, to join the ranks of the League at once in some capacity whilst they had the opportunity.

The resolution was seconded by Sir George Fardell, M.P., supported by the Rev. Prebendary Ridgeway, Vicar of Christ Church, Lancaster Gate, who, in his capacity as President of the League of Mercy for Paddington, spoke eloquently on the importance of requisitioning the personal service of those already interested in hospitals.

This concluded the speaking, and the company enjoyed the remainder of the evening in the grounds, listening to the strains of the band of the Royal Engineers, which, by permission of Major-General Sir Thomas Fraser, K.C.B., executed a delightful programme. A number of the guests did not leave The Lodge until they had responded to the appeals to join the League and signed their names on the roll in token thereof.

